# A case of COVID‐19‐associated mucormycosis due to *Lichtheimia ramosa*

**DOI:** 10.1002/jcla.24895

**Published:** 2023-05-14

**Authors:** Shima Aboutalebian, Mahzad Erami, Mansooreh Momen‐Heravi, Arezoo Charsizadeh, Seyed Jamal Hashemi Hezaveh, Amir Hassan Matini, Amir Hossein Ahsaniarani, Mojtaba Fakhrehi, Hossein Mirhendi

**Affiliations:** ^1^ Department of Medical Parasitology and Mycology, School of Medicine Isfahan University of Medical Sciences Isfahan Iran; ^2^ Mycology Reference Laboratory, Research Core Facilities Laboratory Isfahan University of Medical Sciences Isfahan Iran; ^3^ Department of Infectious Disease, School of Medicine, Shahid Beheshti Hospital Kashan University of Medical Sciences Kashan Iran; ^4^ Department of Medical Parasitology and Mycology, School of Public Health Tehran University of Medical Sciences Tehran Iran; ^5^ Immunology, Asthma, and Allergy Research Institute Tehran University of Medical Sciences Tehran Iran; ^6^ Department of Pathology and Histology, School of Medicine, Shahid Beheshti Hospital Kashan University of Medical Sciences Kashan Iran; ^7^ Department of Otorhinolaryngology, School of Medicine, Matini Hospital Kashan University of Medical Sciences Kashan Iran

**Keywords:** COVID‐19, diabetes, *Lichtheimia ramosa*, mucormycosis, rhinosinusitis

## Abstract

**Background:**

Mucormycosis is a life‐threatening invasive fungal infection in immunocompromised and COVID‐19 patients.

**Case Report:**

Here, we report a fatal rhino‐orbito‐cerebral mucormycosis caused by *Lichtheimia ramosa,* in a 79‐year‐old diabetic female. She was initially admitted to the hospital for COVID‐19 infection and received broad‐spectrum antibiotics and corticosteroids. After 1 month, she was admitted again because of persistent headaches and decreased right eye movement when the computed tomography scan showed mucosal thickening and opacification of paranasal sinuses. Microbiological investigations, including culture and direct microscopy, and histopathological findings confirmed the diagnosis of proven mucormycosis. The isolated causal agent was identified as *Lichtheimia ramosa* by sequencing the entire ITS region of nuclear ribosomal DNA. Despite surgical debridement and administration of liposomal amphotericin B 5 mg/kg/day, the patient's level of consciousness suddenly deteriorated; she was intubated and mechanically ventilated in the ICU and died on the same day.

**Conclusion:**

To our knowledge, this is the first worldwide case of COVID‐19‐associated rhino‐orbito‐cerebral mucormycosis due to *Lichtheimia ramosa*.

## 1 INTRODUCTION

Mucormycosis is a rare opportunistic fungal infection that mainly affects individuals with predisposing conditions such as uncontrolled diabetes mellitus, neutropenia, hematological malignancies, receiving steroids, and organ transplantation.^1^, ^2^ The sinuses, brain, and lungs are the primary sites affected by mucormycosis. The major clinical forms of the infection are rhino‐orbito‐cerebral and pulmonary and rarely gastrointestinal, cutaneous, and disseminated.^3^

The unprecedented mucormycosis outbreaks in India and some other parts of the world have highlighted COVID‐19 as a significant risk factor^4^; however, COVID‐19‐associated mucormycosis (CAM) or post‐COVID‐19 infections have been reported from almost all over the world. The most clinical manifestation of mucormycosis in COVID‐19 patients has been reported as rhinosinusitis and rhino‐orbito‐cerebral forms.^2^ The intertwined relationship between COVID‐19 and mucormycosis worsens infection extension and mortality rates. Although diabetes has been the most frequent risk factor for mucormycosis before the COVID‐19 outbreak, in diabetic COVID‐19 patients and even nondiabetic ones, long‐ or even short‐term use of corticosteroids has often been associated with the deadly upsurge of mucormycosis.^4^, ^5^, ^6^ Mucormycosis is an invasive fungal infection caused by the members of the order Mucorales. *Rhizopus oryzae* is the most common causative agent responsible for nearly 60% of infections globally, followed by the other *Rhizopus* species, *Mucor* species, and *Lichtheimia* (formerly *Absidia*, *Mycocladus*) species. *Rhizopus* species are mostly recorded in patients with diabetes mellitus, while *Lichtheimia* infections were primarily associated with hematological malignancies and presented as severe cutaneous and pulmonary infections.^6^, ^7^

Here, we report a post‐COVID‐19 rhino‐orbito‐cerebral mucormycosis in a 79‐year‐old diabetic woman. As far as we know, this is the first case of rhino‐orbito‐cerebral CAM caused by *Lichtheimia ramosa* worldwide, which could be an emerging Mucoralean fungus.

## 2 CASE REPORT

On August 19, 2021, a 79‐year‐old female with a history of diabetes mellitus, ischemic heart disease, and hypertension was hospitalized with a possible diagnosis of COVID‐19 in Shahid Beheshti Hospital, Kashan, Iran. Physical examination of her head and neck, chest, heart, abdomen, and limbs showed no abnormal findings. Nasopharyngeal and oropharyngeal swab samples became positive in real‐time reverse transcription polymerase chain reaction (rt‐RT‐PCR) test performed by Light Cycler 96 system (Roche) to detect SARS‐CoV‐2 targeting N and RdRp genes (Pishtaz Teb).^6^, ^8^ The patient was managed with antibiotics for suspected bacterial superinfection, and with corticosteroids, including prednisolone (125 mg/day) and dexamethasone (8 mg intravenously/daily) for a week until PCR for SARS‐CoV‐2 was negative.

**FIGURE 1:**
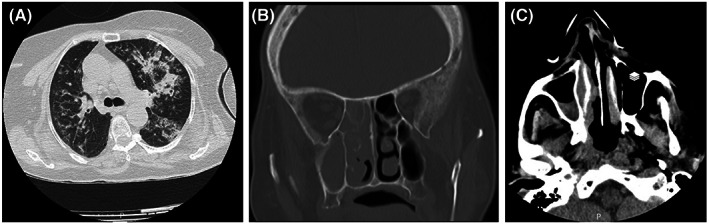
(A) Chest CT scan demonstrating multiple peripheral patchy grand glass and consolidation with air bronchogram, highly suggestive of COVID‐19 pneumonia. (B and C) Paranasal CT scan showing mucosal thickening and opacification in right maxilla, frontal and ethmoid sinuses, and right and left sphenoids.

The patient was admitted again to the hospital 1 month later, presenting right facial swelling and deviation of the angle of the mouth to the left, complete ophthalmoplegia, no perception of light, and ptosis along with the right eye. She suffered from headaches, cough, dyspnea, and weakness. Physical examination revealed a body temperature of 37.3°C, blood pressure of 111/60 mm Hg, pulse rate of 79 beats per minute, respiratory rate of 20 breaths per minute, and oxygen saturation of 86% while the patient was breathing ambient air. The laboratory tests revealed an erythrocyte sedimentation rate (ESR) of 16 mm in the 1st h and a mild increase in CRP (26 mg/L). Both oropharyngeal rt‐RT‐PCR for COVID‐19 and blood/urine culture for bacteria and fungi were negative. The chest computed tomography (CT) scan demonstrated multiple peripheral patchy grand glass and consolidation with air bronchogram in the left lower lobe (LLL) and right middle lobe (RML), highly suggestive of COVID‐19 pneumonia (Figure 1A). With the possibility of post‐COVID‐19 bacterial superinfection, the antibiotics azithromycin, meropenem, and vancomycin were started. Because of persistent headache and considering the possibility of mucormycosis, a paranasal computed tomography scan was performed that showed mucosal thickening and opacification in the right maxilla, frontal and ethmoid sinuses, and right and left sphenoid (Figure 1B,C). Gradually, the right eye movement decreased. After consulting with an ENT specialist, the patient was undergoing endoscopic surgery. Anterior and posterior ethmoidectomy and sphenoidotomy were performed, and the necrotic tissue was resected. With increased hypoxia and progressive reduction of SPO2, the patient was subjected to mechanical ventilation and transferred to ICU.

**FIGURE 2:**
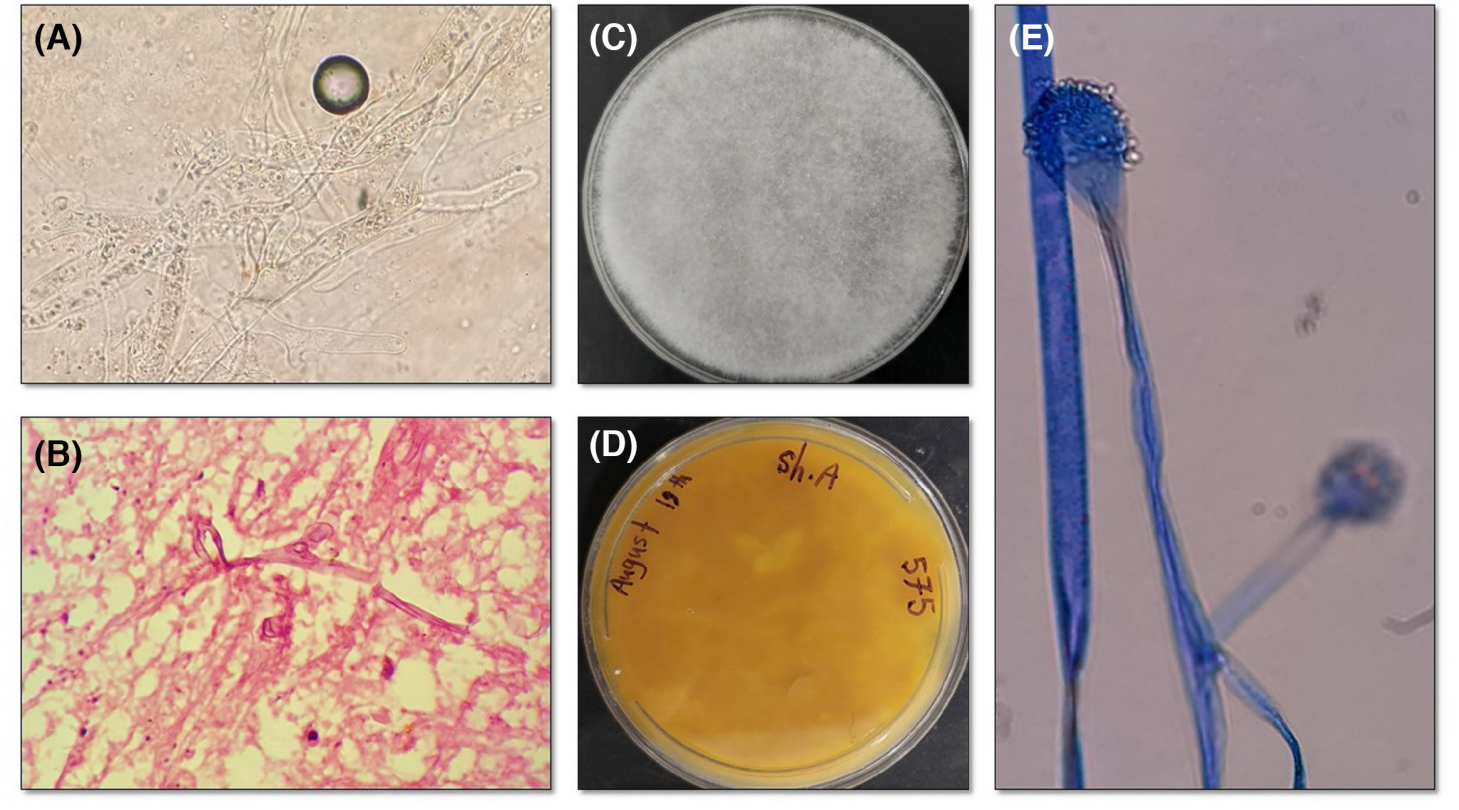
(A) Direct microscopic examination using 20% KOH revealing broad aseptate hyaline hyphae with right‐angle branching. (B) Histopathologic examination with H&E staining showing infiltration of acute and chronic inflammatory cells including lymphoplasma cells and neutrophils and fungal aseptate hyphae. (C and D) Surface and reverse isolated colony after 4 days of incubation. (E) Microscopic morphology of the isolated fungus microcolony in slide culture.

The resected tissue specimen was subjected to direct microscopic examination with 20% potassium hydroxide (KOH) and histopathological preparation stained with hematoxylin and eosin (H&E). The direct microscopy demonstrated broad aseptate hyaline hyphae with right‐angle branching (Figure 2A). The histopathology showed infiltration of acute and chronic inflammatory cells, including lymphoplasma cells and neutrophils, as well as fungal aseptate hyphae (Figure 2B). These conditions suggest a proven invasive mucormycosis; therefore, intravenous liposomal amphotericin B (5 mg/kg/day) was initiated. Despite antifungal and antibacterial treatment and debridement during two endoscopic surgeries, the patient's condition deteriorated. Finally, after 15 days, on September 2, 2021, the patient died due to respiratory failure, hemodynamic instability, and mucormycosis with the invasion of the orbit and probably the brain.

Parts of the samples were inoculated at three points of Sabouraud dextrose agar (Merck) plates containing 50 mg/L chloramphenicol and incubated at 25 and 37°C. After 4 days of incubation, colonies with coarse woolly gray surfaces rapidly covering agar plate with fluff resembling gray cotton candy (Figure 2C), and with a white reverse, indicative of a *mucoralean* fungus, were grown (Figure 2D). Slide culture and staining with aniline blue revealed the microscopic morphology suggestive of *Lichtheimia* species (Figure 2E). DNA of the colony was extracted using glass bead extraction followed by phenol‐chloroform purification method as described previously,^9^ the entire ITS region of nuclear ribosomal DNA was PCR‐amplified using the universal primer pairs ITS1 and ITS4,^10^ and the products were purified and sequenced (Core Facilities Laboratory) by forward primer. After analysis of the sequence by BLAST (https://blast.ncbi.nlm.nih.gov/Blast.cgi), the isolate was identified as *Lichtheimia ramosa*, with 99.87% sequence identity (only one adenine (A) deletion). The sequence was deposited in GenBank under accession number ON239992. Finally, the clinical and laboratory findings confirmed the diagnosis of the disease as rhino‐orbito‐cerebral mucormycosis due to *Lichtheimia ramosa*.

Antifungal susceptibility testing (AFST) of the isolate was tested for amphotericin B, *isavuconazole*, posaconazole, and itraconazole according to CLSI M38‐A2. As per the protocol, RPMI‐1640 medium with 3‐(N‐morpholino) propanesulfonic acid and without bicarbonate, an inoculum of 0.4–5 × 104 CFU/mL fungal spores, and incubation at 35°C were used. The results were read visually 24 h after incubation, and the lowest concentration at which the fungal growth was completely inhibited was recorded as the minimum inhibitory concentration (MIC) value. *C. parapsilosis* ATCC 22019 was used as a quality control strain. AFST of the isolate against amphotericin B, *isavuconazole*, posaconazole, and itraconazole revealed MIC values of 0.25, 1, 0.0313, and 0.0313 μg/mL, respectively.

## 3 DISCUSSION

Mucormycosis is one of the most severe infections due to its rapid insidious onset, fast progression, and extremely high mortality.^11^ Before the beginning of COVID‐19, the overall mortality rate of mucormycosis was estimated to be 54%, and the disseminated form was about 96% fatal.^12^, ^13^, ^14^ Viral infections are not among the classical risk factors of mucormycosis; however, severe respiratory viral infections, including severe influenza and COVID‐19, may cause elevated inflammatory cytokine blood levels that lead to immune dysregulation and defects in the function of neutrophils and macrophages as well as abnormalities in the number of granulocytes and monocytes. In our case, uncontrolled blood sugar and the use of corticosteroids were the main underlying predisposing factors that led to COVID‐19‐associated mucormycosis. Diabetes causes acidosis environment and immune imbalances allowing Mucorales to thrive and predisposing patients to severe COVID‐19.^4^, ^5^, ^6^ Hyperglycemia causes phagocyte malfunction, poor chemotaxis, and inefficient intracellular killing. Furthermore, epithelial damage, especially in the basement membrane, often caused by diabetes or chemotherapy, altogether makes diabetic COVID‐19 patients highly susceptible to mucormycosis.^4^, ^5^, ^6^

Some hospitalized COVID‐19 patients, especially those caused by the delta variant of the virus having severe courses of the disease, received heavy doses of steroids (dexamethasone, methylprednisolone, and tocilizumab) as a part of treatment. Corticosteroids suppress macrophages and neutrophils to reduce the inflammation in the lung and decrease the damages that may happen in the body due to the cytokine storm. Steroids may act as a double‐edged sword, simultaneously reducing both inflammation and the activity of the immune system, thereby creating a favorable environment for other opportunistic diseases. Moreover, corticosteroids trigger the uncontrolled release of sugar and can cause hyperglycemia even in healthy individuals (steroid‐induced diabetes), resulting in ketoacidosis, which in diabetic patients lead to augmentation of immunosuppression,^6^, ^15^ therefore, could increase the risk of developing invasive fungal infections including mucormycosis.^4^, ^6^, ^15^ Vaezi et al. reviewed the incidence and potential risk factors for developing mucormycosis in 25 years between 1990 and 2015 in Iran. Diabetes (47.9%) was the most common underlying condition in Iranian patients, followed by patients who had transplants (22.4%), leukemia (6.1%), and neutropenia (2.0%).^16^

Saprophytic fungi of the order Mucorales, including *Rhizopus*, *Lichtheimia*, *Mucor*, *Rhizomucor*, *Apophysomyces*, *Saksenaea*, *Cunninghamella*, *Cokeromyces, Actinomucor*, and *Syncephalastrum* species, are the causative agents of mucormycosis; the first three species account for 70–80% of all cases.^17^ *Rhizopus oryzae*, the most common cause of infection, is associated with the rhino‐orbito‐cerebral form and is mainly predisposed by ketoacidosis. *Lichtheimia* species represent the second and third most common cause of mucormycosis in Europe and worldwide, respectively,^18^ and are primarily associated with corticosteroid use and hematological malignancies.^7^, ^18^ The frequency of *Lichtheimia* infections in Europe is significantly higher than in the USA and Asia.^19^ Overall, the epidemiology of mucormycosis has shown different patterns worldwide.^20^

According to morpho/physiological and molecular data, *Lichtheimia* contains at least five species: *L. corymbifera*, *L. ramosa*, *L. ornata*, *L. hyalospora*, and *L. sphaerocystis*, which are isolated from various body sites including the respiratory tract, paranasal sinuses, brain, heart, blood, and wound. *Lichtheimia ramosa* (syn. *L. hongkongensis*, formerly *Absidia idahoensis*) is the most common pathogenic *Lichtheimia* species, currently regarded as an emerging pathogen associated with pulmonary and cutaneous infections^18^ and has been increased from 5% to 19% in the last decade.^21^ It is reported in a variety of patients with different underlying diseases.^22^ As they are very similar in morphology, a recent study has claimed that a significant proportion of *L. corymbifera* isolated from human infections is indeed *L. ramosa*.^23^ *L. ramosa* has most often been isolated from cases of cutaneous and pulmonary mucormycosis^24^; indeed, only three cases (including the current case) of sinonasal or rhino‐orbito‐cerebral infections due to *L. ramosa* have been documented, all of them died. Woo et al.^25^ described a rhinocerebral mucormycosis due to *L. ramosa* in a 65‐year‐old Chinese man with hepatitis B, liver cancer, liver transplant, and GVHD.^25^ Houaida et al.^26^ reported a case of *L. ramosa* infection resulting in the sinonasal invasion in a 59‐year‐old Tunisian woman with diabetes and acute myeloid leukemia. Herein, we report the third fatal case of sinusitis due to *L. ramosa* (the first COVID‐19‐associated case) in a patient who recovered from COVID‐19 infection and had almost all critical risk factors for the development of mucormycosis, including diabetes, corticosteroid use, and hypertension.

Accurate species identification of human pathogenic Mucorales is challenging, mainly due to the frequent lack of diagnostic morphological features, particularly the fact that germination and production of fungal spores are highly dependent on culture media and incubation conditions. The recent guidelines published by the Clinical and Laboratory Standards Institute recommend using ITS sequencing as the best method for identifying species within the Mucorales.^27^ Thus, we used this strategy to identify the Mucorales we isolated from the patients.

Like other mucormycosis, the treatment of *L. ramosa* infection involves the combination of antifungal treatment by amphotericin B and posaconazole as well as surgical debridement. The MICs for amphotericin B were significantly lower for *L. ramosa* than *L. corymbifera*.^28^

## 4 CONCLUSION

This is the first case of rhino‐orbito‐cerebral CAM due to *Lichtheimia ramosa*. As a fungal coinfection in the COVID‐19 pandemic, mucormycosis is one of the most alarming concerns; therefore, clinicians should use corticosteroids for patients wisely, adopt early and precise diagnosis of cases, check glycemic levels, and apply timely treatment or/and surgical operations.

## AUTHOR CONTRIBUTIONS

Mahzad Erami and Shima Aboutalebian performed all the experiments and participated in data collection. Shima Aboutalebian and Arezoo Charsizadeh drafted the manuscript. Shima Aboutalebian analyzed and interpreted the data. Mansooreh Momen‐Heravi, Seyed Jamal Hashemi Hezaveh, Mojtaba Fakhrehi, Amir Hossein Ahsaniarani, and Amir Hassan Matini participated in collecting the clinical isolate and data collection. Hossein Mirhendi supervised all parts of the study and critical review of the manuscript. All the authors approved the final version of the manuscript.

## CONFLICT OF INTEREST STATEMENT

The authors declare that the research was conducted in the absence of any commercial or financial relationships that could be construed as a potential conflict of interest.

## Data Availability

The data supporting the findings of this study are openly available in NCBI at http://www.ncbi.nlm.nih.gov/blast.
